# Supplementary material for: Few-femtosecond passage of conical intersections in the benzene cation
Source: Nat Commun. 2017 Oct 18;8:1018. doi: 10.1038/s41467-017-01133-y (PMC5715116; doi:10.1038/s41467-017-01133-y)
Supplement: Supplementary file 1 — Supplementary Information [file 41467_2017_1133_MOESM1_ESM.pdf]

SUPPLEMENTARY NOTE 1

The dimensionless effective nuclear coordinate Q_{eff} is a suitable linear combination of all normal mode coordinates exhibiting a substantial first-order coupling to the electronic motion (the C-C stretching mode ν_2 and the Jahn-Teller active normal modes ν_{16}-ν_{18}) and is chosen to yield a balanced description of the relevant low-energy conical intersections.

SUPPLEMENTARY NOTE 2

These are the fragments $C_3H_3^+$, $C_4H_4^+$, $C_5H_3^+$, $C_6H_4^+$ and $C_6H_5^+$.

SUPPLEMENTARY NOTE 3

For the fragment $C_4H_2^+$ a slope of $m^{C_4H_2^+}_{\Delta t=0\text{ fs}}$=$2.0 \pm 0.1$ was found for time-overlap, whereas the S/N level of the data recorded at $\Delta t = 80$ fs does not permit quantifying a slope m.

SUPPLEMENTARY NOTE 4

Note also that $\tilde{D}$-state population, which stems from decay of the $\tilde{E}$ state, might have a higher detection efficiency than directly extreme-ultraviolet (XUV) populated $\tilde{D}$-state population. The total internal energy is larger in the first case, which might lead to more efficient formation of the signature fragments. Such an effect would however not affect the timescales extracted in this work.

SUPPLEMENTARY NOTE 5: STATE-SPECIFIC CATION-CATION TRANSITIONS IN THE PROBE STEP

Here we investigate the cation-cation transitions in the two-photon experimental probe step in more detail. For simplicity, the calculation is based on the equilibrium geometry of the neutral benzene molecule. First, the cationic eigenstates of the benzene molecule have been calculated using the algebraic diagrammatic construction (ADC) scheme [1–3] for representing the Green's function, namely the non-Dyson ADC(3) scheme [4] correct up to the third order of perturbation theory. The ionisation spectrum up to 20 eV is shown in Supplementary Figure 1 a). Next, to consider the two-photon transitions between the cationic states, the matrix elements of the square of the dipole moment operator $\hat{\vec{D}}$ have been computed using the general representation of a one-body operator within the many-body basis of the cationic Hamiltonian (see Ref. [5]). The approximation is standard for narrow transition line nonresonant multiphoton processes [6] and justified by the large spectral bandwidth of the ultrashort visible/near-infrared (VIS/NIR) pulses used here. It assumes that the intermediate states reached by the first VIS/NIR probe photon form a full basis. The corresponding transition probabilities are obtained by squaring the matrix elements $\langle i| \hat{\vec{D}}^2 |f\rangle$. For transitions originating from the $\tilde{D}$ and $\tilde{E}$ electronic states, the transition probabilities are plotted in arbitrary units in Supplementary Figure 1 b), at the energetic positions of the final state. Black poles represent cation-cation transitions originating from the $\tilde{D}$ component states, while green poles represent such transitions originating from the $\tilde{E}$ state.

Also plotted in Supplementary Figure 1 b) are the relevant two-photon probe spectra for ionisation from the $\tilde{D}$ state (black) and for ionisation from the $\tilde{E}$ state (green). They are derived by convoluting the experimentally measured frequency-domain spectrum of the VIS/NIR pulse with itself and shifting it by the calculated $\tilde{D}$ state energy (14.4 eV, dashed vertical black line) and the $\tilde{E}$ state energy (15.0 eV, dashed vertical green line), respectively. Relevant for the state-specific probing efficiency are the transition probabilities that fall within the respective two-photon probe spectrum. As can be seen from Supplementary Figure 1 b), there are five target states within the two-photon probe spectrum for $\tilde{D}$-state probing (black), four of which are located between 17.7 and 18.2 eV, while there are only two target states within the two-photon probe spectrum for $\tilde{E}$-state probing (green). Importantly, the target states for $\tilde{E}$-state probing, reached by the high-energy tail of the two-photon spectrum, are however located much higher in energy, between 19.0 and 19.2 eV.

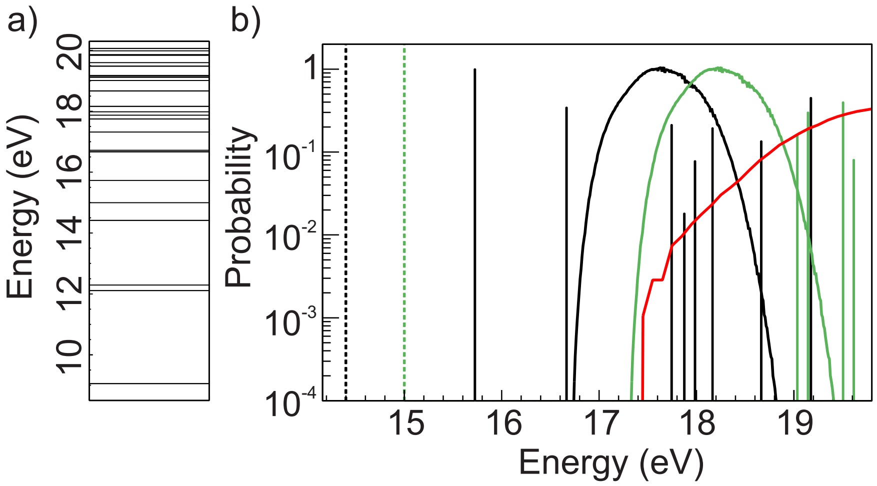

Supplementary Figure 1: **Computational investigation of the probe step.** **a)** Computed energy levels of benzene cationic states in the energy range up to 20 eV. **b)** Computed cation-cation transition probabilities for two-photon excitation, $\Gamma = | \langle i | \mathrm{D}^2 | \mathrm{f} \rangle |^2$, in arbitrary units, plotted as sticks at the energetic position of the final state, for initial states $\tilde{D}$ (black) and $\tilde{E}$ (green). Smooth black and green curves represent the relevant two-photon probe spectrum, originating from the $\tilde{D}$- and $\tilde{E}$-state energies (black/green dashed vertical lines). The red curve depicts the relative $C_4H_3^+$ ion yield for XUV ionisation of neutral benzene, reproduced from Ref. [7]. The analysis indicates that $\tilde{E}$-state probing is slightly preferred over $\tilde{D}$-state probing, since higher lying target states in the range of 19.0-19.2 eV are populated, where the relative $C_4H_3^+$ fragment abundance is larger.

In principle, to obtain the efficiency of probing population in the $\tilde{D}$ and $\tilde{E}$ states via $C_4H_3^+$ fragment creation, the respective transition probabilities plotted in Supplementary Figure 1 b) need to be added up for all energies, weighted with the respective two-photon spectral intensity and with the final state-specific probability to form the signature $C_4H_3^+$ fragments. Unfortunately, the latter probability is not directly available. However, what has been measured with synchrotron radiation by Holland *et al.* is the photon energy-dependent relative ion abundance upon ionisation of neutral benzene [7]. From their study, we reproduce the energy-dependent relative yield of $C_4H_3^+$ plotted as a red line in Supplementary Figure 1 b) in arbitrary units. From the threshold at 17.5 eV onwards, the yield of this fragment is strongly increasing with energy in the relevant range. Higher lying final states thus have a significantly increased probability of forming the signature fragment. In particular, this is true for the target states between 19.0 and 19.2 eV reached by $\tilde{E}$-state probing.

It is clear from Supplementary Figure 1 b) that a convolution of transition probabilities, photon spectra and fragment yield depends very sensitively on the quantitative details of each of those. In particular, the relative probing efficiency of the $\tilde{D}$ and $\tilde{E}$ state depends critically on the exact computed values of the energies of the target states. The estimated uncertainties of these energies are typically a few hundred meV and up to about 1 eV for satellite states [8]. Given these uncertainties, the limitation due to considering only the equilibrium geometry and the vibrational ground state, and the approximation of the state-specific probability to form the signature fragment, we cannot predict the relative probing efficiencies fully quantitatively. A naive convolution of the transition probabilities, the two-photon spectral intensities and the fragment yield as depicted in Supplementary Figure 1 b), however, suggests that $\tilde{E}$-state probing is slightly preferred over $\tilde{D}$-state probing in our experimental cation-cation transition probe scheme. This is consistent with the good agreement of our experimental data with a model incorporating a relative probing efficiency of $\mathrm{Eff}_{\tilde{E}}/\mathrm{Eff}_{\tilde{D}} = 2$ (see inset Fig. 4 main text).

SUPPLEMENTARY NOTE 6: MULTI-CONFIGURATION TIME-DEPENDENT HARTREE (MCTDH) CALCULATION FOR DIFFERENT INITIAL STATE CONDITIONS

In our MCTDH simulations, five component electronic states have been treated: the $\tilde{E}$, $\tilde{D}_{x,y}$ and $\tilde{B}_{x,y}$ states (see methods). To construct the theoretical 'yield curve' of Fig. 4 (see main text), we consider separately three different initial conditions at zero time. These are: (1) imposed pure $\tilde{E}$-state population, (2) imposed pure $\tilde{D}_x$-state population, (3) imposed pure $\tilde{D}_y$-state population. The two components $\tilde{D}_x$ and $\tilde{D}_y$ overall show the same behaviour as expected and are combined for the present analysis. The dynamical calculations are usually performed in the

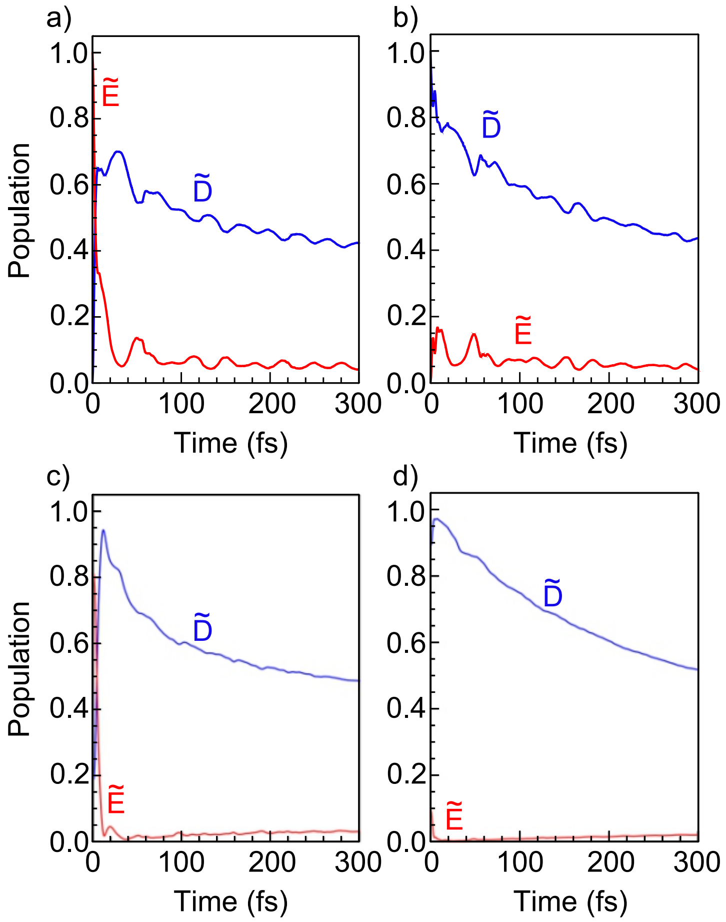

Supplementary Figure 2: **Details of the MCTDH simulation.** Time-dependent diabatic (panels **a)**, **b)**) and adiabatic (panels **c)**, **d)**) populations of the $\tilde{E}$ and $\tilde{D}$ states derived from the MCTDH calculation for an initial pure population at zero time, of the $\tilde{E}$ state (panels a), c)) and the $\tilde{D}$ state (panels b), d)), respectively. Populations of $\tilde{E}$, $\tilde{D}$ and $\tilde{B}$ (not shown) add up to 1 for all time delays. The populations plotted here underlie the summation described in the main text, which produces the diabatic and adiabatic yield curves shown in Fig. 4 (main text).

diabatic representation, therefore the populations obtained in the simulations are typically the diabatic ones. These are shown in the two upper panels of Supplementary Figure 2. In Supplementary Figure 2 a) we plot the obtained time-dependent population for the $\tilde{E}$ and $\tilde{D}$ diabatic states, for an imposed pure $\tilde{E}$-state population at zero time. As can be seen, the initial $\tilde{E}$-state population very rapidly transitions to the $\tilde{D}$ state, on a timescale of about 10 fs. The $\tilde{D}$ state then decays on a much slower timescale and populates the $\tilde{B}$ component states. In Supplementary Figure 2 b), the same populations are shown for an imposed initial pure $\tilde{D}$-state population, comprised half of $\tilde{D}_x$ and half of $\tilde{D}_y$. While a small fraction of the population is transferred to the $\tilde{E}$ state very rapidly, most of the $\tilde{D}$-state population decays on a slower timescale to the $\tilde{B}$ state. The populations in Supplementary Figure 2 a) and b) are summed as described in the main text, to obtain the dashed yield curve of Fig. 4 (main text). The oscillations observed in the diabatic populations depicted in Supplementary Figure 2 a) match the 33 fs period of the C-C stretching mode. The fact that the oscillations are not seen in the experiment motivated us to compute the adiabatic populations as well. We note here that such calculations are computationally much more expensive (by about one order of magnitude) than the diabatic ones, and that they would have been impossible at the time of Ref. [9]. In order to obtain the adiabatic populations from the propagated wave functions in the diabatic basis, one has to generate projection operators onto the adiabatic states. In the MCTDH code, the computation of the latter requires to invoke the primitive product grid. This is costly and brings the current calculation to the limits of our computational resources. The adiabatic populations are shown in the lower two panels of Supplementary Figure 2 for initial population of the $\tilde{E}$ (panel c)) and the $\tilde{D}$ (panel d)) state. One sees immediately that the oscillations which characterised the diabatic populations are no longer visible. These features likely emerge from variations of the adiabatic-to-diabatic mixing angle and are

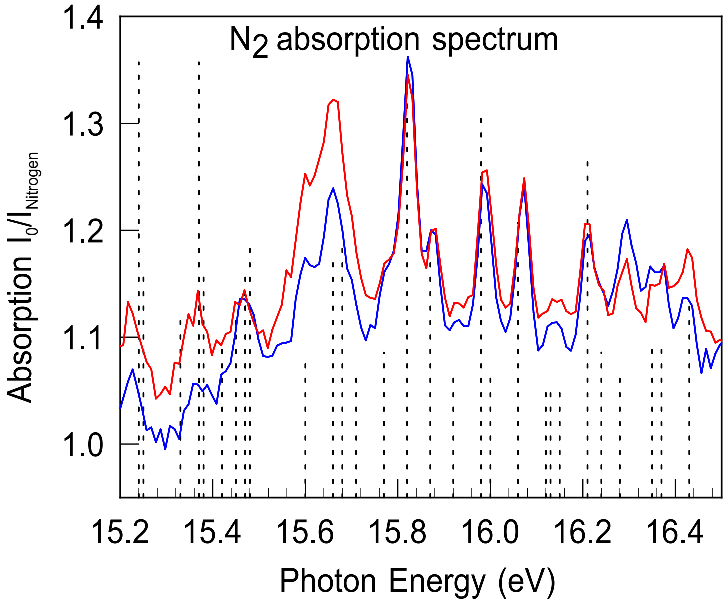

Supplementary Figure 3: **Calibration of the XUV spectrum.** Two different nitrogen absorption spectra are shown (blue and red lines). The spectra were calibrated by comparing to literature values (dotted black lines) taken from [11].

considered to be an artefact of the diabatic theory, observed also in previous two-state calculations [10]. The adiabatic yield curve is obtained by summing the populations in Supplementary Figure 2 c) and d) as explained in the main text and shown as solid line in Fig. 4 (main text).

SUPPLEMENTARY NOTE 7: CALIBRATION OF THE XUV SPECTRUM

In order to characterise which electronic states play a role in this study, the XUV spectrum is precisely calibrated by measuring the absorption of XUV photons by molecular nitrogen. Nitrogen is supplied through a repeller-integrated pulsed valve in order to achieve the high molecular densities required to observe significant absorption. The absorption spectrum was measured by first recording the XUV spectrum over 100 laser shots without and then for 100 shots with nitrogen gas in the supply line. The ratio of these two spectra $\mathrm{A} = \mathrm{I}_0/\mathrm{I}_{\mathrm{N}_2}$ is displayed in Supplementary Figure 3 for different measurements (red and blue lines). Nitrogen shows distinct absorption lines with considerable absorption cross-section down to ≈ 14.5 eV photon energy, covering the entire range displayed in Supplementary Figure 3. These absorption lines have been previously assigned in the literature and originate from Rydberg series converging to the ionic ground state $X^2\Sigma_g^+$ and the first excited ionic state $A^2\Pi_u$. The energy positions and approximate relative oscillator strengths of these features are displayed as dotted lines in Supplementary Figure 3 and were extracted from Ref. [11]. The XUV spectrum is calibrated by assuming a linear relation between camera pixel and photon energy axis. This approach is not accurate for the entire spectral range of the XUV spectrometer, but is sufficient for a single high harmonic as can be seen by the good agreement between the measured features and reference energy positions. We estimate the overall precision of our energy calibration to be better than 50 meV.

SUPPLEMENTARY REFERENCES

[1] Schirmer, J., Cederbaum, L. S. & Walter, O. New approach to the one-particle Greens-function for finite Fermi systems. *Phys. Rev. A* **28**, 1237–1259 (1983).

[2] Cederbaum, L. S. *Encyclopedia of Computational Chemistry*, chap. Green's Functions and Propagators for Chemistry, p.1202–1211 (Wiley, Chichester, 1998).

[3] Trofimov, A. & Schirmer, J. Molecular ionization energies and ground- and ionic-state properties using a non-Dyson electron propagator approach. *J. Chem. Phys.* **123**, 144115 (2005).
[4] Schirmer, J., Trofimov, A. B. & Steller, G. A non-Dyson third-order approximation scheme for the electron propagator. *J. Chem. Phys.* **109**, 4734–4744 (1998).
[5] Schirmer, J. & Trofimov, A. B. Intermediate state representation approach to physical properties of electronically excited molecules. *J. Chem. Phys.* **120**, 11449–11464 (2004).
[6] Meshulach, D. & Silberberg, Y. Coherent quantum control of multiphoton transitions by shaped ultrashort optical pulses. *Phys. Rev. A* **60**, 1287–1292 (1999).
[7] Holland, D. *et al.* A time-of-flight mass spectrometry study of the fragmentation of valence shell ionised benzene. *Int. J. Mass Spectrom.* **220**, 31 – 51 (2002).
[8] Deleuze, M. S., Trofimov, A. B. & Cederbaum, L. S. Valence one-electron and shake-up ionization bands of polycyclic aromatic hydrocarbons. I. Benzene, naphthalene, anthracene, naphthacene, and pentacene. *J. Chem. Phys.* **115**, 5859–5882 (2001).
[9] Köppel, H., Döscher, M., Bâldea, I., Meyer, H.-D. & Szalay, P. G. Multistate vibronic interactions in the benzene radical cation. II. Quantum dynamical simulations. *J. Chem. Phys.* **117**, 2657–2671 (2002).
[10] Manthe, U. & Köppel, H. Dynamics on potential-energy surfaces with a conical intersection - adiabatic, intermediate, and diabatic behavior. *J. Chem. Phys.* **93**, 1658–1669 (1990).
[11] Carter, V. L. High-Resolution N_2 Absorption Study from 730 to 980 Å. *J. Chem. Phys.* **56**, 4195–4205 (1972).
